# Global Harmonization of Biosimilar Development by Overcoming Existing Differences in Regional Regulatory Requirements - Outcomes of a Descriptive Review

**DOI:** 10.1007/s43441-024-00740-4

**Published:** 2025-01-16

**Authors:** Thomas M. Kirchlechner, Hillel P. Cohen

**Affiliations:** 1 Sandoz GmbH, Biochemiestrasse 10, Kundl, 6250 Austria; 2 Highland Park, NJ USA

**Keywords:** Biosimilar, Regulatory harmonization, Ethnic sensitivity, Animal toxicology studies, Bridging studies, Streamlining biosimilar development

## Abstract

Global harmonization of biosimilar developmental requirements will facilitate development leading to increased patient and societal benefits. However, there are several technical and regulatory hurdles that must be addressed to harmonize the regulatory requirements in different countries and regions. At times, there is a requirement for use of locally sourced reference product, forcing biosimilar developers to repeat analytical or clinical comparability studies against reference product batches sourced from within a given country. While most health authorities no longer require comparative animal toxicology studies of the proposed biosimilar and reference product, these are still required in several countries, forcing biosimilar companies to conduct such studies or risk non-approval of their product. At times, different health authorities request different clinical study designs. In some jurisdictions there is a requirement to generate clinical data in local ethnic populations. Some health authorities require a hybrid label that combines clinical data from the reference biologic and the biosimilar, in the patient leaflet. Recommendations are provided to address each of these hurdles to facilitate global regulatory harmonization of biosimilars. Overcoming these barriers will ultimately increase patient access to these medicines in all regions while providing financial relief to healthcare systems.

## Introduction

Laws, regulations and guidelines for the development, review, and post-approval regulation of biosimilars have been implemented in many countries. The European Union (EU) pioneered biosimilar regulations by establishing a new regulatory pathway resulting in a new product type in 2004, based on the fundamental understanding that biosimilars are biological medicines Most other regions and countries developed equivalent requirements in the 2010s. Initial regulations were relatively conservative as would be expected for a new class of therapeutic drugs and a new development pathway that comes with a new evidentiary and regulatory review process. While many health authorities around the world have looked to the EU as model for the biosimilar pathway, health authorities in several countries have taken an individual approach for establishing some elements of biosimilarity. Although not being a regulatory body, the World Health Authorization (WHO) issues recommendations and had recognized the need for global harmonization of biosimilar regulations early, leading to publication of their first biosimilar guidance in 2009 [1]. The WHO guidance provided a globally applicable reference document that was particularly useful for health authorities who had not begun or were in the process to design their own biosimilar legal framework. Some health authorities have adopted WHO guidelines as their own requirements, so in such countries, WHO recommendations have a regulatory impact. WHO continues to support and consult with health authorities around the world [2] and extensively updated its biosimilar guidance in 2022 [3]. Despite these efforts, there are still jurisdictions that treat biosimilars as if they are generic products and other jurisdictions where they are treated as novel drugs.

Many national or regional health authorities have clarified in their local biosimilar regulations that they expect a candidate biosimilar product to comply with ICH guidelines that are applicable to *originator* biological products. This is reassuring since it helps ensure that biosimilars are held to the same quality standards as their reference products and it facilitates harmonization among these countries and regions.

The biosimilar regulatory environment continues to evolve as health authorities assess the data generated to support approval of multiple biosimilars over the years to see which studies provide meaningful data and which studies are extraneous [4–6]. As a result of this ongoing analysis some but not all health authorities are modifying their requirements to further streamline development based on latest scientific knowledge, and by clarifying the most appropriate and useful data for regulatory assessment of biosimilars.

For the biosimilar industry, global harmonization of biosimilar regulatory requirements would enable a single biosimilar development program that addresses all regulatory requirements in all regions and countries. This would also benefit health care providers worldwide who want to be able to rely on a single robust standard for all. The impact of divergent regulatory requirements delays development, increases development costs, and ultimately impacts patients for whom the benefit of biosimilar availability is delayed or in some counties may even be blocked. It is possible that there may be isolated cases of local health authorities establishing divergent requirements or legislation to protect their domestic pharmaceutical industry, but this is only speculative.

The need for a single globally harmonized and streamlined biosimilar development has become acute because it is now apparent that the complex development requirements and high biosimilar development costs are limiting development of biosimilars to many biologics that are no longer protected by patents or other forms of exclusivity. Estimates have been made that, by 2027, up to 55% of biological drugs for which patent protection has expired will have no biosimilar competition [7]. Absent biosimilar competition limits access for many patients to the high-cost biological drugs and places a significant cost burden on healthcare systems. It also means that additional sources of supply are not developed, which could be important for supply security in the long run. In addition, local patient access is also impacted by local intellectual property issues as well as marketing considerations whereby a company may elect not to market a product in a given country for commercial reasons. There are also policy incentives to support biosimilar uptake as well as tactics employed by manufacturers of reference products to maintain market share, all of which impact biosimilar utilization. However, these factors should not impede global development and regulatory harmonization efforts.

Global regulatory convergence for biosimilar development should be possible, but the first step is to identify similarities and differences. Prior reviews have identified some differences between health authority expectations and requirements for biosimilars and also discuss potential harmonization [8, 34, 43–50], We have reexamined the similarities and differences by investigating the individual national regulatory requirements and guidelines relevant to biosimilars in more than 70 countries and regions as of July 2024. We included emerging countries with health authorities that have less experience with biosimilars as well as countries with health authorities that have extensive experience with biosimilars. Similarities and differences were identified. We categorize these differences, discuss their implications, and provide suggestions on how global regulatory harmonization of biosimilars can be improved.

## Methods

Biosimilar regulations and guidelines from 68 countries and seven regions were identified by searching the websites of individual health authorities and pharmaceutical industry trade associations (including e.g. the Association for Accessible Medicines, the Biosimilars Forum, the International Generics and Biosimilars Association, Medicines for Europe). We also examined databases maintained by the Generics and Biosimilars Initiative and the Center for Biosimilars. We observed that some countries have a single biosimilar guidance document while others have multiple guidelines on different aspects of biosimilarity. A separate legal framework for biosimilars is in place for some countries or regions. We observed that some countries do not have distinct biosimilar requirements or guidance documents, e.g. Russia, Laos, Myanmar, Cambodia, French-speaking West- and Central African countries. All countries identified that have at least one biosimilar guidance or a legal framework are listed in Table 1.

**Table 1:** Scope of national and regional biosimilar guidelines included in this review

Geography	National guidelines	Regional guidelines
Africa	Algeria, Angola, Egypt, Ethiopia, Ghana, Kenya, Morocco, Nigeria, Rwanda, South-Africa, Sudan, Tanzania, Tunisia, Uganda, Zambia, Zimbabwe	East-African Community (EAC), Southern-African Development Community (SADC)
Asia Pacific	Australia, Bangladesh, China, Hong Kong, India, Indonesia, Japan, Malaysia, New Zealand, Pakistan, Philippines, Singapore, South Korea, Taiwan, Thailand, Vietnam	Association of Southeast Asian Nations (ASEAN)
Community of Independent States (CIS)	Kazakhstan	Eurasian Economic Union (EAEU)
Europe	Belarus, Bosnia-Herzegovina, Georgia, Great Britain, Russia, Serbia, Switzerland, Turkey, Ukraine	European Medicines Agency (EMA)
Latin America	Argentina, Bolivia, Brazil, Chile, Colombia, Costa Rica, Cuba, Dominican Republic, Ecuador, Guatemala, Mexico, Panama, Peru, Trinidad&Tobago, Uruguay, Venezuela	Central America & Caribbean (CAC)
Middle East	Bahrain, Iran, Iraq, Israel, Jordan, Lebanon, Saudi-Arabia, United Arab Emirates	Gulf Cooperation Council (GCC)
North America	Canada, USA	

The obtained documents were individually analyzed to ascertain the general analytical, pharmacological and clinical requirements used to establish safety and efficacy prior to local approval. Special attention was paid to the requirements for the following development and registration parameters:

- Analytical and clinical comparability against *locally* sourced reference product.
- Animal toxicology studies.
- Country-specific design of the pivotal clinical study.
- A requirement for clinical data from a *local* patient population.
- A format of the product label that deviates from the generic label approach (see Results section for definitions).

In addition, a manual, unstructured review was conducted of English-language scientific publications authored by regulators from health authorities from any country, covering the time period of Jan 2019 to June 2024. All such publications were examined to determine if they contained recommendations for the development, review or pharmacovigilance of biosimilars.

## Results

All countries and regions surveyed accept the concept that biosimilars are developed to match the key characteristics of the reference biologic, with analytical assays forming the initial level of evidence. The analytical comparisons contain chemical, biochemical, biophysical, and biological function (bioassay) methodologies [46]. There may be differences in the rigor and extent of the analytical comparisons from country-to-country, but that is a reflection of the health authorities and not of the concept itself.

In the overwhelming majority of countries, biosimilars are directly compared to the reference biologic in all key studies. A small number of countries have another pathway in which the copy biological drug is developed to match the features of the reference biologic, but the two are not compared in a head-to-head manner. In our opinion, it is possible that these copy biologics may be safe and effective, but they should not be considered as biosimilars [3].

An examination of all available biosimilar regulations and guidelines from countries and regions listed in Table 1 revealed that there were five areas in which regulatory variability currently exists from country-to-country or region (Table 2). These are:

1. Analytical and clinical comparability against locally sourced reference product.
2. Animal toxicology studies.
3. Design of the pivotal clinical study.
4. A requirement for clinical data from a local patient population.
5. The format of the product label (or monograph, as it may be termed locally).

All countries and regions exhibiting regulatory divergence in at least one of the five areas mentioned above, are presented in Table 2. The analysis did not differentiate between local laws or non-binding local regulatory guidelines. Divergence by category is discussed in separate sections below. Divergence in pivotal study design is discussed below in a separate section but is not summarized in Table 2 because the diversity observed in clinical study design does not lend itself to presentation in a tabular format.

**Table 2:** Overview of Regulatory requirements for biosimilar products in 21 countries and regions. Analytical and clinical comparability are presented separately in the table by showing analytical in column 2 and clinical comparability, combined with local clinical data requirements, in column 4. Divergent pivotal clinical study design requirements are not included in this table

	Analytical bridge required: Local reference product to reference product obtained from another country	Animal toxicology studies required	Clinical bridge to local reference product or local clinical data required	Hybrid label used that contains data from both the reference product and biosimilar
***Country/Region***				
**Europe**				
1. European Union	Y	N	Y	N
2. Switzerland	N	N	N	N
3. United Kingdom	N	N	N	N
***North America***				
4. Canada	N	N	N	N
5. United States	Y	N	Y	N
***Latin America***				
6. Argentina	N	Y	N	N
7. Brazil	C	Y	N	Y
8. Dominican Republic	N	Y	N	
9. Guatemala	N	N	N	Y
10. Mexico	N	N	N	N
***Asia Pacific***				
11. Australia	Y	N	N	Y
12. China	Y	Y	Y	Y
13. India	N	N	Y^2^	N
14. Japan	Y	N	N^1^	Y
15. Korea	Y	Y	Y^2^	N
16. New Zealand	C	N	N	Y
17. Taiwan	C	N	Y	N
18. Thailand	N	Y	N	N
19. Singapore	C	N	N	Y
***Middle East***				
20. Iran	N	Y	N	N
21. Iraq	N	Y	N	N

Y = Yes, required; N = No, not required; C = conditional

^1^= not required if product is ethnically insensitive

^2^= local clinical data can either be provided as part of the marketing authorization application, e.g. comparative pharmacokinetic trial or participation in global clinical trial, or post-approval in a post-marketing safety (PMS) study

### Analytical and Clinical Comparability against Locally Sourced Reference Product

The requirement for a local reference product as the comparator product was incorporated into many early biosimilar requirements and guidances in many countries. It is based on concerns that a given brand product might differ in some important parameters in different countries. This has been seen with some generic chemical drugs but was only a theoretical concern for biological drugs. With time has come a recognition that such differences have not been detected with biological drugs. The EMA, Health Canada and several other health authorities are now willing to accept non-local reference products. In addition, this has become a de facto requirement if a sponsor seeks approval of a biosimilar in a country in which the originator was approved but in which it is no longer marketed for commercial reasons. Biosimilars are developed on the basis of what is approved locally and not what is marketed locally [34].

From a purely scientific perspective, an analytical comparability exercise against the EU- and/or the US-sourced reference product is sufficient to enable a *global* biosimilar development because there is only a single set of clinical data supporting the reference product in all regions of the world. Any local deviations, whether they are related to differences in specifications or perhaps in manufacturing sites, have been studied and justified by the manufacturer of the reference product and had to have been proven to be not clinically meaningful or else the differences would not have been licensed. It is the manufacturer of the reference product who has scientifically proven that their product has no clinically meaningful differences globally. Theoretically, material sourced from *any* country with a stringent Regulatory authority that follows ICH guidelines and WHO recommendations could be considered to be a suitable *global* comparator product [8].

As a practical matter, this science-based argument is not yet accepted in many countries. As a result, several health authorities require an analytical comparison of reference product sourced from their country or region with any other reference product used in pivotal analytical and clinical studies. Our analysis revealed that South Korea [9], China [10], Japan [11], Australia [12], but also EU countries [13] and the U.S [14]. do not accept comparator products sourced outside their jurisdiction but require an analytical bridging study against *locally* sourced batches of the same comparator brand. As a result, these countries cannot be covered by a single, global biosimilar development but require additional development efforts to generate analytical, and in some cases such as the US, additional PK data comparing the biosimilar product and/or batches of the global comparator against batches of the locally sourced reference product. As outlined in the subsequent section on local clinical data, South Korea, China, and Japan also require local clinical data in addition to analytical comparability against the locally sourced reference product.

There are some countries that will accept analytical data generated with non-local reference product but require that locally sourced reference product be used for comparative clinical studies (Table 2).

Countries that conditionally accept comparator products sourced outside their jurisdiction for analytical comparisons, e.g., if sameness of comparator manufacturing site can be proven by public domain information (same site supplying foreign and local jurisdiction), include Brazil [15], New Zealand [16], Singapore [17] and Taiwan [18]. These countries can often be covered by a single, global biosimilar development program by adding literature-based evidence proving the sameness of reference product obtained from different countries.

### Animal Toxicology Studies

Initial biosimilar guidelines mandated that animal toxicology testing of biosimilars must be conducted and included in biosimilar license applications. The EMA, US FDA, Japanese PMDA, Australian TGA, Health Canada as well as the WHO made this recommendation based in part on the development paradigm of originator drugs where animal toxicology testing is conducted to make certain that drug candidates are sufficiently safe for human clinical studies. It was also thought that comparative animal toxicology testing would provide a safety net by ensuring that the animal toxicology testing results of a proposed biosimilar were not different and were certainly not worse than those of the reference product.

It has become apparent that both biosimilars and their reference products are commonly highly immunogenic in animals as one would expect when human or humanized proteins are administered to another species. Due to the scientific advancement of biosimilar drug development and experience gained over time, a paradigm shift has taken place over the past decade that gradually replaced animal toxicology testing with biological in vitro functional assays based on the fact that in vitro bioassays are very often more specific and sensitive to detect differences between the biosimilar and the reference product than toxicology studies conducted in animals. The removal of animal toxicology testing in the US via the FDA Modernization Act of 2022 [54] was made in recognition of the fact that animal toxicology testing of biosimilars had not proven to provide value in biosimilar development to the degree that the FDA and EMA routinely granted waivers so that biosimilars could be developed without such studies. The term “animal toxicology testing” was replaced in US legislation with the term “non-clinical testing” which admittedly can include animal testing but more importantly also includes in vitro bioassays [51, 52]. Removal of such studies is also consistent with efforts to minimize animal testing that is of limited or no utility. Consequently, waivers of animal toxicology testing have routinely been provided upon request. In many countries this requirement has been removed completely from biosimilar requirements and guidelines.

Despite this global trend to move away from obligatory animal toxicology studies, at least 8 countries in Latin America, Africa, Middle East, and Asia Pacific still mandate animal toxicology testing. These include Brazil [15], Argentina [19], Dominican Republic [20], Iran [21], Iraq [22], South Korea [9], Thailand [23], and China [10].

### Divergent Requirements for Clinical Studies Including Study Design

Optimal biosimilar development programs would enable a biosimilar developer to conduct a single set of studies that are suitable and acceptable in all jurisdictions. Unfortunately, this is not always the case for biosimilars as at times different health authorities request different clinical study designs for the confirmatory clinical study. For example, some health authorities may request the use of traditional clinical efficacy endpoints in a comparative phase-3 like efficacy and safety trial, whereas other health authorities will accept or may even prefer pharmacodynamic (PD) surrogate biomarkers instead, if such biomarkers are available. There may also be requirements to monitor patient safety after initiation of biosimilar therapy at different time intervals or to follow patient safety for different lengths of time.

While most discussions between sponsors and health authorities are not public, several examples are available in the public domain. The U.S. FDA and the EMA had different requirements for the design of the confirmatory clinical study for the Sandoz filgrastim Biosimilar (Zarzio^®^/Zarxio^®^, filgrastim). EMA specified that given the analytical data package and the nature of the molecule, pivotal clinical confirmation of biosimilarity could be obtained via a set of pharmacokinetic comparison studies. It is worth noting that filgrastim is a relatively simple biologic medicines with a good PD marker. EMA approved Zarzio^®^ in 2009. In contrast, the FDA required a phase-3 style efficacy and safety design to provide clinical confirmation. The Sandoz filgrastim was the very first biosimilar to be approved in the U.S. in 2015, and it is possible that under those circumstances the FDA was being especially cautious.

Another example of how regulatory decision-making on biosimilars can evolve, is the marketing authorization request for CT-P13 (Inflectra^®^/Remsima^®^, infliximab). Both the EMA and FDA licensed this product for all indications for which the reference biologic had been approved, including inflammatory bowel disease (IBD) indications (ulcerative colitis, Crohn’s disease), based on extrapolation from an efficacy and safety study in patients with active rheumatoid arthritis (RA) and a pharmacokinetic study in patients with ankylosing spondylitis (AS). However, Health Canada was not willing to approve IBD for the biosimilar based on extrapolation, although they did allow extrapolation to the other indications. Subsequent studies conducted with the biosimilar in IBD indications revealed that the clinical performance of the biosimilar matched that of the reference biologic, supporting *post facto* the decisions of the EMA and FDA. We have not seen other subsequent examples where extrapolation of indications was not granted by a major health authority for a biosimilar candidate, but it remains possible.

Recent studies point to the feasibility to develop biosimilars without clinical comparative efficacy or PD trials while maintaining the regulatory robustness [4, 5, 24, 25] The Medicines and Healthcare products Regulatory Agency of the UK (MHRA) enabled such a streamlined development in 2021 [6]. Its biosimilar guidance states that in most cases, a comparative efficacy trial may not be necessary. EMA released a concept paper on application of a tailored clinical approach in biosimilar development in 2024 [26]. A review by EU regulators of marketing applications for 36 monoclonal antibodies and antibody derived fusion proteins evaluated by EMA revealed that clinical comparative confirmation was never a decisive criterion. They concluded that a sufficiently robust analytical/functional similarity package, together with a pharmacokinetics (PK) trial capturing data on safety and immunogenicity would be sufficient for the purpose of regulatory decision making for biosimilars. However, at present there is no clear roadmap on comparative efficacy studies for developers seeking to pursue a single global development program.

Safety follow-up is another area of divergence among health authorities. Some health authorities require six months of safety follow-up for a given biosimilar after receipt of the last dose for the primary endpoint, while others require 12 months. In practical terms, a biosimilar developer must default to the longer time interval, which delays submission and approval in those countries that require the longer follow-up.

### Local Clinical data Required in some Jurisdictions

Biosimilar developers attempt to develop a single pivotal clinical study design that is acceptable globally, often default to the most conservative requirements. If Good Clinical Practice (GCP) standards are met, most health authorities do not require patient enrollment in specific countries or in specific ethnic populations. However, there are some health authorities that require additional clinical data generated in their local populations.

China [27], Japan [11], and Taiwan [42] require local clinical data for initial approval of drugs. For biosimilars a bridging pharmacokinetics study of the local population to the pivotal clinical trial population is often specified. However, Japan’s MHLW published a new Q&A for biosimilars on 25 January 2024, enabling the acceptance of non-Japanese patient data when the product is ethnically insensitive [28]. South Korea and India require local clinical data, but it can be provided after initial authorization by means of post-marketing surveillance. South Korea requires data from 400 local patients [29] and India requires data from 200 local patients [30]. To our knowledge no analysis of reference product or biosimilar data has ever detected a difference in those different populations. Further research to explore this may be helpful to convince health authorities that such studies are not necessary for biosimilars.

### Label Format

Two approaches are seen globally in the content of the product labels (or product monographs, as it they may be called locally) for biosimilars. In most countries, the product label is based on the product label of the reference product. However, there are some countries in which the product label is a hybrid that contains data from studies conducted with both the reference product and the biosimilar.

The EMA and the U.S. FDA and many other health authorities mandate that a biosimilar medicine should have product labels based on the reference product in a manner that is analogous to small molecule generic drugs. A product label is intended to provide healthcare providers with the key PK, PD, safety, and efficacy of a given molecule. PK, PD, safety, and efficacy of the reference biologic was established by the original development program of the reference biologic. The purpose of the biosimilar development program is to establish biosimilarity, and not to re-establish PK, PD, safety or efficacy. As a result, the biosimilar clinical development program does not provide any new clinically relevant information that is not already known from the originator molecule [31, 32].

It is possible that the approved indications, presentations, or administration device of a biosimilar medicine are different from the reference biologic medicine. If that is the case, the label will reflect this information. For example, a biosimilar may have fewer indications at time of initial approval because the reference biologic may have some indications that are still covered by remaining patents or exclusivity. In addition, biosimilars need not have all presentations as that of the reference biologic.

Australia, Brazil, China, Guatemala, Japan, New Zealand, and Singapore each utilize hybrid labels for biosimilars, although the nature of biosimilar data included in these hybrid labels varies between these countries. For example, in Brazil [33], the following data are expected in the biosimilar label:

- “Pharmacokinetic and pharmacodynamic study results, which were obtained in the comparative studies between the biosimilar and the comparator biological product.”
- “Efficacy results of the comparator biological product should be described, mentioning its commercial name and the results obtained in the comparative studies between the biosimilar and the comparator biological product.”

## Discussion

Global adoption of a single set of standards and regulatory requirements for biosimilars is clearly an aspirational goal that may be difficult to meet. However, it is potentially useful even if met only in part. The first step for global harmonization is to identify areas of divergence so that those can be addressed.

Examples of required **analytical and clinical comparability against a locally sourced reference product** have been identified. From a scientific perspective a single global biosimilar development program with a single set of comparability data should be acceptable, given that the local requirements have been completed by the reference product and by demonstrating biosimilarity to that reference product, the biosimilar can rely on these outcomes.

In our opinion, global alignment of biosimilar requirements is hampered by two factors. Firstly, some national health authorities seek to be viewed as making independent decisions. Secondly, data is often not shared between health authorities. To help address these contributing factors, we propose increased information exchange of these health authorities. We acknowledge that a comprehensive information exchange of this nature may be challenging because it will likely require negotiations and agreements at the ministerial level between sovereign nations.

The concept of a global comparator for biosimilar development is a current topic of discussion [8, 34].

It appears that initial steps are being taken to streamline biosimilar clinical development. The WHO biosimilar guidelines were revised in 2022 [3] in a manner that opens the possibility of eliminating comparative efficacy studies and PD studies for some biosimilars. Another discussion of potential streamlining of biosimilar development took place in 2023 when the Biosimilars Working Group of the International Pharmaceutical Regulators Program (IPRP) hosted a virtual workshop on “Reevaluating the need for comparative clinical efficacy studies in biosimilar development”, which was attended by over 20 regulators globally [35]. The IPRP efforts in general are an important step in global regulatory harmonization.

The regulatory science initiatives undertaken under the US Biosimilars User Fee Act III Commitment Letter as a part of the Regulatory Science Pilot Program have the potential to identify opportunities that could simplify and accelerate biosimilar development. However they will take time to be completed and should be viewed as complementary to the analysis and recommendations presented in this publication.

Once studies with the reference biologic have already demonstrated that there is no ethnic sensitivity in patient response, there is no longer any value in conducting clinical studies with a specific local population. A globally aligned clinical development program for biosimilars with a single set of studies for all countries and regions would avoid unnecessary clinical studies and save patient resources and time.

**Local clinical data** is, in our opinion, not warranted for biosimilars once studies with the reference biologic have demonstrated that there is no ethnic sensitivity in patient response, Numerous reviews and surveys have already shown that the efficacy of parenteral biologics is generally not impacted by patient ethnicity [36, 37]. Studies have found that patient metabolisms towards monoclonal antibodies and other complex proteins are not subject to the same variability as are often observed with small molecule drugs. Only a few cases of weak ethnic sensitivity for biological drugs have been described in the literature. Nevertheless, in these situations, there was no need to adjust the posology or treatment regimen in any of those cases [38–40]. If ethnic sensitivity of a particular biological product does exist, it would already have been detected and studied with the reference product prior to approval in that country or region. This goes back to the fundamentals of biosimilar extrapolation: Since it is the same therapeutic molecule, the biosimilar will exhibit the same efficacy and safety profile as the originator drug in all indications *and* patient populations. We recommend that waivers be granted for biosimilars as the local laws and regulations were promulgated when there was a theoretical concern related to originator drugs that does not exist for biosimilars.

Applicants who pursue a global development program must fulfill the requirements of *all* countries. If one country requests **animal toxicology studies**, the applicant must decide between conducting an animal toxicology study of questionable value or foregoing licensure in that country. Global harmonization by elimination of animal toxicology testing for biosimilars would speed biosimilar development without impacting patient safety and at the same time enhance animal welfare.

Inclusion of the biosimilar clinical information into the label results in a **hybrid label** which gives the incorrect impression that this clinical data was pivotal for product approval, whereas the bedrock of biosimilar approval relies on comparative analytics. Furthermore, the analytical data package supporting a biosimilar is commonly more robust when compared to that of a reference biologic but at the same time the clinical data package is often smaller. Inclusion of comparative clinical data without a discussion of supportive analytical data may give the incorrect impression that there is less data supporting a biosimilar when compared to the reference biologics.

Global harmonization of the label format to use a format based on the reference product will create a level playing field for all biological drugs, whether they are reference biologics or biosimilar. It is noteworthy that the Canadian Regulatory authority has recently updated their biosimilar labeling guideline and now adopted a generic-style label for biosimilars such that used in the EU and the U.S, and no longer uses a hybrid label format [41]. It is important to acknowledge that even reference products often have different labeling in various regions; they too do not have a universal label. Biosimilars are developed based on the most extensive label of the originator that is available globally with comprehensive data packages that address all potential indications. Of course, a local application may need to be tailored to match the local label of the reference product. Local label harmonization is beyond the scope of this publication as it often depends on local intellectual property considerations. But challenges in local label harmonization should not preclude global biosimilar development and global regulatory harmonization.

It is common for reference products to be in short supply or unavailable in many smaller markets for a variety of reasons, including commercial considerations. Conceivably, global harmonization of biosimilar requirements could encourage companies that are currently focusing on local biosimilar markets to seek approval in additional countries and regions thereby increasing both supply and patient access.

Global harmonization of biosimilar development has potential to both accelerate patient access and may enable biosimilars to be developed to reference products that have limited market capitalization. But it is important to note that there are other factors that impact patient access to biosimilars that can promote or limit biosimilar use.

There have been several policy practices undertaken to promote biosimilar use. In some regions of Germany, quotas have been instituted to ensure that biosimilars are prescribed to a predefined portion of patients [55]. In the UK, benefit-sharing has been utilized by which healthcare networks, including hospitals, physicians and payers reinvest savings from biosimilars into other areas of the healthcare system to improve the quality of health care and to increase patients’ access to innovative services and medicines [56]. All Canadian provinces and territories have implemented mandatory switching programs so that for some molecules, new patients start on biosimilars, and existing patients are switched from reference products to biosimilars [57].

But there are also steps being pursued by reference product manufacturers to maintain market share for their products, especially in the US. When biosimilar infliximab became available in the US, the manufacturer of reference infliximab bundled the reference product with other drugs and medical supplies so that if a purchaser selected a biosimilar in place of the reference product they risked losing discounts on all products included in the bundle [58]. After introduction of biosimilars of infliximab, pegfilgrastim, rituximab and trastuzumab in the US the manufacturers of the reference products increased the rebates that they paid to pharmacy benefit managers (PBMs) to maintain a preferred position for the reference products on the PBM formularies. This incentivized PBM use of reference products, but patients did not benefit because patient co-pays are based on the list price of the drug and not the net price after rebates [59]. Globally, “product-hopping” is a tactic being employed by manufacturers of reference products whereby attempts are made to move new or existing patients from a reference product that is subject to biosimilar competition to another product with marginally better efficacy that is still under patent protection. As an example, the manufacturer of reference adalimumab is seeking to supplant adalimumab with risankizumab-rzaa or upadacitinib [60]. Taken as a whole, global regulatory harmonization has potential to accelerate biosimilar development and might enable development of biosimilars to more molecules. But this cannot be viewed in a vacuum because promotive policies and disruptive tactics play an important role as well.

## Conclusions

This descriptive review reveals many commonalities from country-to-country in the requirements to develop biosimilars. However, there are also country-specific differences in some biosimilar regulatory requirements. In our opinion, steps can be taken to globally harmonize biosimilar requirements. The fact that reference products are themselves developed based on a single global development program with all subsequent modifications linked back to the original product, is the basis of the concept of global comparator. Acceptance of the concept of a global comparator would eliminate the need for 3-way bridging pharmacokinetic studies that compare local reference product, foreign-sourced reference product and the proposed biosimilar. It may also be possible to eliminate the need to conduct analytical comparisons of local and foreign-sourced reference product. A single harmonized clinical study design is possible based on sound scientific principles. It is not necessary to conduct clinical studies on a local population, nor is it necessary or appropriate to conduct animal toxicology studies. Opportunities exist to streamline global biosimilar development, including elimination of comparative clinical efficacy and comparative PD studies. The label format for biosimilars is already harmonized between the EU, US, and many other major markets, and should be accepted elsewhere as well.

In general, there are global regulatory harmonization challenges for all drugs. But the lack of global harmonization for biosimilars is impeding development and ultimately patient access to this new class of biological drugs. The biosimilar market is still fragile and long-term success is not a given.

Many health authorities are small and lack the capacity of larger health authorities as are found in the US and many EU countries. Global regulatory harmonization will be challenging and take time because divergent health authorities will need to accept recommendations for global harmonization of biosimilars. To some extent, reliance procedures can be a help in this process, particularly if it is combined with a learning process. The suggestions made in this publication may be challenging to implement given the history of global regulatory divergence, but we view our recommendations as aspirational. Indeed, the recent efforts of the International Pharmaceutical Regulators Program (IPRP) group to evaluate the need to conduct comparative efficacy testing of biosimilars has demonstrated that global regulatory harmonization effort can be undertaken but also that such efforts will require time to reach consensus and then get implemented [53].

In the long term the global harmonization of some or all biosimilar requirements and guidelines will speed and lower the cost of development leading to better patient access to these biological drugs and will help make healthcare more affordable without compromising safety and effectiveness. The sustainability of products in a national market is equally important but separate, and ultimately will ensure that patients have access to these important medication alternatives.

## Data Availability

No datasets were generated or analysed during the current study.

## References

[1] World Health Organization (WHO). 2009. Guidelines on evaluation of similar biotherapeutic products (SBPs) https://cdn.who.int/media/docs/default-source/biologicals/trs_977_annex_2.pdf?sfvrsn=e2389a69_3_download=true. [Accessed Feb 2024].

[2] World Health Organization (WHO). 2019–2023. Five-year plan to help build effective and efficient regulatory systems. https://iris.who.int/bitstream/handle/10665/332461/WHO-MVP-RHT-2019.01-eng.pdf?sequence=1 [Accessed Feb 2024].

[3] World Health Organization (WHO). 2022. Guidelines on evaluation of biosimilars. Replacement of Annex 2 of WHO Technical Report Series, No. 977. https://www.who.int/publications/m/item/guidelines-on-evaluation-of-biosimilars. [Accessed Feb 2024].

[4] Guillen E, Ekman N, Barry S, Weise M, Wolff-Holz E. 2023. A Data Driven Approach to Support Tailored Clinical Programs for Biosimilar Monoclonal Antibodies. Clin Pharmacol Ther. 20.,113(1), 108–123. 10.1002/cpt.2785. Erratum in: Clin Pharmacol Ther. 2023, 113(4): 933. https://pubmed.ncbi.nlm.nih.gov/36546547/ [Accessed Feb 2024]. 10.1002/cpt.2785 36546547

[5] Kirsch-Stefan N, Guillen E, Ekman N, Barry S, Knippel V, Killalea S, Weise M, Wolff-Holz E. Do the outcomes of clinical efficacy trials Matter in Regulatory decision-making for Biosimilars? BioDrugs. 2023;37(6):855–71. 10.1007/s40259-023-00631-4. https://pubmed.ncbi.nlm.nih.gov/37831324/. [Accessed Feb 2024]. 37831324 10.1007/s40259-023-00631-4 PMC10581956

[6] Medicines and Healthcare products Regulatory Agency (MHRA), UK. 2022. Guidance on the licensing of biosimilar products. https://www.gov.uk/government/publications/guidance-on-the-licensing-of-biosimilar-products/guidance-on-the-licensing-of-biosimilar-products [Accessed Feb 2024].

[7] IQVIA. 2023. Assessing the biosimilar void. https://www.iqvia.com/insights/the-iqvia-institute/reports-and-publications/reports/assessing-the-biosimilar-void [Accessed Feb 2024].

[8] Cohen HP, Turner M, McCabe D, Woollett G. Future evolution of Biosimilar Development by application of current science and available evidence: the developer’s perspective. BioDrugs. 2023;37(5):583–93. 10.1007/s40259-023-00619-0 37542600 10.1007/s40259-023-00619-0 PMC10432323

[9] Ministry of Food and Drug Safety (MFDS), South-Korea. 2022. Biosimilar product evaluation guideline. https://www.mfds.go.kr/eng/wpge/m_37/de011024l001.do [Accessed Feb 2024].

[10] National Medicinal Products Administration. (NMPA) China, 2015. China biosimilar guideline.

[11] Pharmaceutical and Medical Device Agency (PMDA), Japan. 2009. Guideline for Ensuring Quality, Safety and Efficacy of Biosimilar Products. https://www.pmda.go.jp/files/000153851.pdf [Accessed Feb 2024].

[12] Therapeutic Goods Adminstraton (TGA), Australia. 2018. Biosimilar medicines regulation. https://www.tga.gov.au/publication/biosimilar-medicines-regulation. [Accessed Feb 2024].

[13] European Medicines Agency (EMA). 2014. Guideline on similar biological medicinal products. https://www.ema.europa.eu/en/documents/scientific-guideline/guideline-similar-biological-medicinal-products-rev1_en.pdf [Accessed Feb 2024].

[14] Food and Drug Administraton (FDA). 2015. Scientific Considerations in Demonstrating Biosimilarity to a Reference Product. https://www.fda.gov/media/82647/download [Accessed Feb 2024].

[15] Agência Nacional de Vigilância Sanitária (ANVISA). 2010. Registration of new biological products and biological products Resolution RDC 55. https://antigo.anvisa.gov.br/documents/10181/2718376/%281%29RDC_55_2010_COMP.pdf [Accessed Feb 2024].

[16] Medsafe New Zealand. 2014. Position on biosimilar medicines. https://www.medsafe.govt.nz/profs/RIss/Medsafe%20position%20on%20biosimilars.pdf [Accessed Feb 2024].

[17] Health Services Authority (HSA). 2016. Singapore Guidance on registration of biosimilar products. https://www.hsa.gov.sg/docs/default-source/hprg/therapeutic-products/guidance-documents/appendix-15_guidance-on-registration-of-biosimilar-products.pdf [Accessed Feb 2024].

[18] Taiwan Food and Drug Administration (Taiwan FDA). 2015. Revised guidelines for the examination and registration of biosimilar medicinal products.

[19] Administración Nacional de Medicamentos, Alimentos y Tecnología Médica (ANMAT). 2011. Requirements for the registration of medicinal specialties of biological origin. Disposición 7075/2011. https://www.argentina.gob.ar/normativa/nacional/disposici%C3%B3n-7075-2011-188580/texto [Accessed Feb 2024].

[20] Dominican Republic Ministry of Health. 2016. Technical Regulation for the Sanitary Registration of Innovative and Non-Innovative Biotechnological Medicines for Human Use in the Dominican Republic. Resolución No. 000018 3/08/2016. https://repositorio.msp.gob.do/handle/123456789/1068 [Accessed Feb 2024].

[21] Food and Drug Organization (FDO). Ministry of Health, Therapeutic & Medical Training, Food and Drug Organization, Islamic Republic of Iran. Registration rules for recombinant biologic drugs and monoclonal antibodies; 2014.

[22] Iraqi Ministry of Health. 2018. National Iraqi guideline on evaluation of biosimilar products.

[23] Thailand Food and Drug Administration (Thai FDA). 2014. Documentary Evidence Required for Biosimilars Registration. Vol. 130, Spec. Part. 174 Government Gazette 4, 2014.

[24] Bielsky MC, Cook A, Wallington A, Exley A, Kauser S, Hay JL, Both L, Brown D. 2020. Streamlined approval of biosimilars: moving on from the confirmatory efficacy trial. Drug Discovery Today. 10.1016/j.drudis.2020.09.006 [Accessed Feb 2024]. 10.1016/j.drudis.2020.09.006 32916269

[25] Schiestl M, Ranganna G, Watson K, Jung B, Roth K, Capsius B, Trieb M, Bias P, Maréchal-Jamil J. The path towards a tailored clinical Biosimilar Development. BioDrugs. 2020;34(3):297–306. 10.1007/s40259-020-00422-1. https://www.ncbi.nlm.nih.gov/pmc/articles/PMC7211192/. [Accessed Feb 2024]. 32266678 10.1007/s40259-020-00422-1 PMC7211192

[26] European Medicines Agency (EMA). 2024. Concept paper for the development of a reflection paper on a tailored clinical approach in biosimilar development. https://www.ema.europa.eu/en/human-regulatory-overview/research-and-development/scientific-guidelines/multidisciplinary-guidelines/multidisciplinary-biosimilar/concept-paper-development-reflection-paper-tailored-clinical-approach-biosimilar-development [Accessed Feb 2024].

[27] National Medicinal Products Administration (NMPA), China. 2020. The clinical technical requirements for authorization of drugs marketed overseas but not currently marketed in China. http://english.nmpa.gov.cn/2020-11/18/c_568155.htm#:~:text=For%20drugs%20marketed%20overseas%20but%20not%20marketed%20in%20China%2C%20technical,Chinese%20patients%2C%20clinical%20safety%20and [Accessed Feb 2024].

[28] Japan Pharma, News. 2024. MHLW No Longer Requires Japanese Comparability Data for Certain Biosimilars: Q&A Update. https://pj.jiho.jp/article/250319 [Accessed Feb 2024].

[29] Sandoz. 2015. Internal communication.

[30] Director Controller Genral of India (DCGI). 2016. Guidelines on Similar Biologics Regulatory Requirements for Marketing Authorization in India, 2016. https://ibkp.dbtindia.gov.in/DBT_Content_Test/CMS/Guidelines/20181115140059519_Guidelines-on-Similar-Biologics-2016.pdf [Accessed Feb 2024]. 10.5731/pdajpst.2012.00886 23035022

[31] European Medicines Agency (EMA). 2023. Biosimilars in the EU – Information guide for healthcare professionals. https://www.ema.europa.eu/system/files/documents/leaflet/biosimilars-eu-information-guide-healthcare-professionals_en_1.pdf [Accessed April 2024].

[32] Sagi S, Anjaneya P, Kalsekar S, Kottke A, Cohen H. Long–term real–world post–approval Safety Data of multiple biosimilars from one marketing–authorization holder after more than 18 years since their first Biosimilar Launch. Drug Saf. 2023;46:1391–404. [Accessed April 2024]. 37902937 10.1007/s40264-023-01371-8 PMC10684613

[33] Agência Nacional de Vigilância Sanitária (ANVISA). 2016. Clarification note 002/2016/GPBIO/CBREM/GGMED/ANVISA. https://www.gov.br/anvisa/pt-br/setorregulado/regularizacao/medicamentos/produtos-biologicos/documentos-orientativos-e-guias/nota-de-esclarecimento-002-de-2016-medicamentos-biologicos.pdf/view [Accessed Feb 2024].

[34] Webster C, Woollett G. 2017. A ‘Global Reference’ Comparator for Biosimilar Development. BioDrugs Aug;31(4):279–286. 10.1007/s40259-017-0227-4. https://pubmed.ncbi.nlm.nih.gov/28526943/ [Accessed June 2024]. 10.1007/s40259-017-0227-4 PMC5541093 28526943

[35] Food and Drug Administration (FDA). 2023. Increasing the Efficiency of Biosimilar Development Programs–Reevaluating the Need for Comparative Clinical Efficacy Studies. https://www.fda.gov/drugs/news-events-human-drugs/increasing-efficiency-biosimilar-development-programs-reevaluating-need-comparative-clinical, [Accessed Feb 2024].

[36] Matsushima S, Huang Y, Suzuki H, Nishinio J, Lloyd P. Ethnic sensitivity assessment - pharmacokinetic comparability between Japanese and non-japanese healthy subjects on selected mAbs. Expert Opin Drug Metab Toxicol. 2015;11(2):179–91. 10.1517/17425255.2015.990438. https://www.tandfonline.com/doi/epdf/10.1517/17425255.2015.990438. [Accessed Feb 2024]. 25519513 10.1517/17425255.2015.990438

[37] Hsieh MM, Everhart JE, Byrd-Holt DD, Tisdale JF, Rodgers GP. 2007. Prevalence of neutropenia in the U.S. population: age, sex, smoking status, and ethnic differences. Ann Intern Med. 3; 146(7):486– 92. 10.7326/0003-4819-146-7-200704030-00004. https://www.acpjournals.org/doi/full/10.7326/0003-4819-146-7-200704030-00004?rfr_dat=cr_pub++0pubmed_url_ver=Z39.88-2003_rfr_id=ori%3Arid%3Acrossref.org [Accessed Feb 2024]. 10.7326/0003-4819-146-7-200704030-00004 17404350

[38] Athalye SN, Baruah DB, Mittra S, Ranpura A, Kumar K, Wolff-Holz E. 2023. Ethnic sensitivity assessments in biosimilar monoclonal antibodies clinical development programmes: necessary or not? Generics and Biosimilars Initiative Journal (GaBI Journal). 2023;12(2): 61– 6. https://gabi-journal.net/ethnic-sensitivity-assessments-in-biosimilar-monoclonal-antibodies-clinical-development-programmes-necessary-or-not.html [Accessed Feb 2024].

[39] Wolff-Holz E, Tiitso K, Vleminckx C, Weise M. Evolution of the EU Biosimilar Framework: past and future. BioDrugs. 2019;33(6):621–34. 10.1007/s40259-019-00377-y. https://link.springer.com/article/10.1007/s40259-019-00377-y. [Accessed Feb 2024]. 31541400 10.1007/s40259-019-00377-y PMC6875146

[40] Wong TY, Cheung CMG, Lai TYY, Chen SJ, Lee WK, Yoon YH, Iida T, Tueckmantel C, Sowade O, Ogura Y. 2019. Efficacy and Safety of Intravitreal Aflibercept and Ranibizumab in Asian Patients with Neovascular Age-Related Macular Degeneration: Subgroup Analyses from the VIEW Trials. Retina, 39(3), 537–547. 10.1097/IAE.0000000000001986. https://www.ncbi.nlm.nih.gov/pmc/articles/PMC6410967/pdf/retina-39-537.pdf [Accessed Feb 2024]. 10.1097/IAE.0000000000001986 PMC6410967 29280937

[41] Health Canada. 2023. Policy statement: Content of product monograph for biosimilar biologic drugs – clinical comparative study results. https://www.canada.ca/en/health-canada/services/drugs-health-products/drug-products/applications-submissions/policies/policy-content-product-monograph-biosimilar-biologic-drugs-clinical-comparative-study-results.html [Accessed Feb 2024].

[42] Taiwan Food and Drug Administration (Taiwan FDA). 2009. Guideline on bridging study - ethnic factors in the acceptability of foreign clinical data. https://www.taiwanclinicaltrials.tw/spotlight/clinical_trial_overview/regulation/faq [Accessed Feb 2024].

[43] Rathore AS, Bhargava A. 2020. Biosimilars in Developed Economies: Overview, Status, and Regulatory Considerations. Regulatory Toxicology and Pharmacology 110; 104525 10.1016/j.yrtph.2019.104525. [Accessed Oct 2024]. 10.1016/j.yrtph.2019.104525 31743691

[44] Cazap E, Jacobs I, McBride A, Popovian R, Sikora K. 2018. Global Acceptance of Biosimilars: Importance of Regulatory Consistency, Education, and Trust. Oncologist Oct;23(10):1188–1198. 10.1634/theoncologist.2017-0671. Epub 2018 May 16. PMID: 29769386; PMCID: PMC6263136. [Accessed Oct 2024]. 10.1634/theoncologist.2017-0671 PMC6263136 29769386

[45] Markus R, Liu J, Ramchandani M, Landa D, Born T, Kaur P. Developing the totality of evidence for Biosimilars: Regulatory considerations and Building confidence for the Healthcare Community. BioDrugs Jun. 2017;31(3):175–87. 10.1007/s40259-017-0218-5. PMID: 28439817; PMCID: PMC5443883. [Accessed Oct 2024]. 10.1007/s40259-017-0218-5 PMC5443883 28439817

[46] Kirchhoff CF, Wang XM, Conlon HD, Anderson S, Ryan AM, Bose A. Biotechnol Bioeng Dec. 2017;114(12):2696–705. 10.1002/bit.26438. Epub 2017 Sep 19. PMID: 28842986; PMCID: PMC5698755. [Accessed Oct 2024]. Biosimilars: Key regulatory considerations and similarity assessment tools. 10.1002/bit.26438 PMC5698755 28842986

[47] Kim H, Alten R, Avedano L, Dignass A, Gomollón F, Greveson K, Halfvarson J, Irving PM, Jahnsen J, Lakatos PL, Lee J, Makri S, Parker B, Peyrin-Biroulet L, Schreiber S, Simoens S, Westhovens R, Danese S, Jeong JH. 2020. The Future of Biosimilars: Maximizing Benefits Across Immune-Mediated Inflammatory Diseases. Drugs Feb;80(2):99–113. doi: 10.1007/s40265-020-01256-5. Erratum in: Drugs. 2020;80(10):1039–1040. 10.1007/s40265-020-01333-9. PMID: 32002851; PMCID: PMC7007415. [Accessed Oct 2024]. 10.1007/s40265-020-01256-5 PMC7007415 32002851

[48] Kang H-N, Thorpe R, Knezevic I, Casas Levano M, Chilufya MB, Chirachanakul P, Chua HM, Dalili D, Foo F, Gao K, Habahbeh S, Hamel H, Kim GH, Perez Rodriguez V, Putri DE, Rodgers J, Savkina M, Semeniuk O, Srivastava S, Tavares Neto J, Wadhwa M, Yamaguchi T. 2021. Regulatory challenges with biosimilars: an update from 20 countries. Ann. N.Y. Acad. Sci., 1491: 42–59. 10.1111/nyas.14522 [Accessed Oct 2024]. 10.1111/nyas.14522 PMC8247359 33222245

[49] González CPV, Muñoz CG. 2020. The controversy around technical standards for similar biotherapeutics: barriers to access and competition? Pharmacoepidemiol Drug Saf. 29: 1518–1522. 10.1002/pds.5100 [Accessed Oct 2024]. 10.1002/pds.5100 32964695

[50] Athalye SN, Mittra S, Ranpura AM. 2023. Biosimilars drug development: time for a paradigm shift? Generics and Biosimilars Initiative Journal (GaBI Journal). 12(1):17–22 10.5639/gabij.2023.1201.005 [Accessed Oct 2024].

[51] Baldrick P. Getting a molecule into the clinic: nonclinical testing and starting dose considerations. Regul Toxicol Pharmacol Oct. 2017;89:95–100. 10.1016/j.yrtph.2017.07.027. Epub 2017 Jul 24. PMID: 28751261. [Accessed Oct 2024]. 10.1016/j.yrtph.2017.07.027 28751261

[52] Niazi SK. 2022. End animal testing for biosimilar approval. Science, Jul 8;377(6602):162–163. 10.1126/science.add4664. Epub 2022 Jul 7. PMID: 35857557. [Accessed Oct 2024]. 10.1126/science.add4664 35857557

[53] IPRP Biosimilars Working Group (BWG). 2024. Workshop Summary Report: increasing the efficiency of Biosimilar Development Programs — Reevaluating the need for comparative Clinical Efficacy Studies. International Pharmaceutical Regulators Programme online publication https://admin.iprp.global/sites/default/files/2024-07/IPRP_BWG_Final%20IPRP%20Scientific%20Workshop%20Summary%20Report_2024_0506.pdf

[54] S.5002–117th Congress. (2021–2022): FDA Modernization Act 2.0| Congress.gov| Library of Congress https://www.congress.gov/bill/117th-congress/senate-bill/5002 [Accessed Oct 2024].

[55] Blankart KE, Arndt F. Physician-level cost control measures and Regional Variation of Biosimilar utilization in Germany. Int J Environ Res Public Health. 2020;17(11):4113. 10.3390/ijerph17114113. PMID: 32526943; PMCID: PMC7313006. [Accessed Dec 2024]. 32526943 10.3390/ijerph17114113 PMC7313006

[56] Barcina Lacosta T, Vulto Arnold G, Turcu-Stiolica A, Huys I, Simoens S. 2022. Qualitative Analysis of the Design and Implementation of Benefit-Sharing Programs for Biologics Across Europe. BioDrugs 217–229, Vol.36 (2).10.1007/s40259-022-00523-z. [Accessed Dec 2024]. 10.1007/s40259-022-00523-z PMC8986662 35303281

[57] Biosimilars Canada. Manitoba Announces Transition to Cost-Saving Biosimilar Medicines - Biosimilars Canada. (August 6, 2024) https://biosimilarscanada.ca/blog/manitoba-announces-transition-to-cost-saving-biosimilar-medicines/#. [Accessed Dec 2024].

[58] US FDA, Docket, FDA-2018-P-3281. Comments from Novartis Services, Inc. in support of Pfizer, Inc.‘s Citizen Petition. (). 2018. https://www.regulations.gov/document/FDA-2018-P-3281-0006 [Accessed Dec 2024].

[59] Mouslim M, Socal MP, Trujillo AJ. Dynamics of biological markets with multiple biosimilar competitors in the United States. Expert Opin Biol Ther Published Online Oct. 2024;9. 10.1080/14712598.2024.2412648. [Accessed Dec 2024]. 10.1080/14712598.2024.2412648 39378044

[60] AbbVie reports third-quarter 2024 financial results. AbbVie. News release, October. 30, 2024. https://investors.abbvie.com/news-releases/news-release-details/abbvie-reports-third-quarter-2024-financial-results [Accessed Dec 2024].

